# Apple consumption is associated with a distinctive microbiota, proteomics and metabolomics profile in the gut of Dawley Sprague rats fed a high-fat diet

**DOI:** 10.1371/journal.pone.0212586

**Published:** 2019-03-14

**Authors:** Jose F. Garcia-Mazcorro, Romina Pedreschi, Jialing Yuan, Jorge R. Kawas, Boon Chew, Scot E. Dowd, Giuliana Noratto

**Affiliations:** 1 Research and Development, MNA de México, San Nicolás de los Garza, Nuevo León, México; 2 Faculty of Veterinary Medicine, Universidad Autónoma de Nuevo León (UANL), General Escobedo, Nuevo León, México; 3 Escuela de Agronomía, Pontificia Universidad Catolica de Valparaiso, La Palma, Chile; 4 School of Food Science, Washington State University, Pullman, Washington, United States of America; 5 Faculty of Agronomy, UANL, General Escobedo, Nuevo León, México; 6 Department of Nutrition and Food Science, Texas A&M University, College Station, Texas, United States of America; 7 Molecular Research LP, Shallowater, Texas, United States of America; National Institute for Agronomic Research, FRANCE

## Abstract

Apples contain bioactive compounds with the potential to alleviate clinical signs associated with obesity, a phenomenon likely related to the composition and function of the gut microbiota. The aim of this study was to investigate the effect of apple supplementation on the fecal microbiota and gut metabolites of Dawley Sprague rats fed a high-fat (HF group) or a low-fat (LF group) diet. The fecal microbiota was examined using 16S marker sequencing targeting the V4 region in a MiSeq instrument (Illumina). With the exception of *Blautia*, which was higher in supplemented rats compared to controls within the LF group, significant differences in fecal microbiota between supplemented rats and controls were only found in the HF group. This suggests that the effect of apple supplementation on the gut microbiota is strongly dependent on the composition of the diet, a phenomenon with potential consequences for obese human patients. Principal Coordinate Analysis of unweighted UniFrac distances revealed a clear strong separation of bacterial communities based on diet (HF and LF, *P* = 0.001, R = 0.69, ANOSIM test) and based on apple supplementation within the HF group, albeit less strongly (*P* = 0.006, R = 0.27, ANOSIM test). No differences were found for fecal SCFAs but proteomics and metabolomics analyses showed differential expression of both proteins and metabolites between supplemented rats and controls in the HF group. The results of this study can guide future explorations of the effect of apple supplementation on human health.

## Introduction

The digestive tract of humans and other mammals is colonized by trillions of bacteria and other microorganisms (known as the gut microbiota) that play a fundamental role in health and disease. Different factors from the host and from the environment are known to be related to the composition and metabolic activities of the gut microbiota but diet is one of the main contributors [1,2]. Importantly, the relationship between diet and the gut microbiota is partly responsible for the health benefits associated with the consumption of certain dietary ingredients or the adoption of particular dietary patterns.

Apples and other plant foods contain bioactive compounds with health-bearing properties. For example, apple phenolics have been shown to possess anti-inflammatory properties in mice [3] and growing evidence suggests that these positive effects are associated with the gut microbiota. Masumoto et al. [4] showed that non-absorbable procyanidins (a class of anti-oxidants flavonoids) from apples can prevent obesity in mice, a phenomenon that was associated with a decrease in the cecum of the Firmicutes:Bacteroidetes ratio and an increase in the abundance of the mucin degrader *Akkermansia*. Another study showed that apple flavonoids are associated with decreases in some inflammation markers and differences in the gut microbiota of healthy mice [5]. Apples are also rich in other bioactive compounds such as pectin, which is a heteropolysaccharide that is considered to act as a prebiotic *in vivo*. Pectin from apples has been shown to promote the presence of anti-inflammatory commensal bacteria such as *Faecalibacterium prausnitzii* [6] and can improve gut barrier function and attenuate metabolic endotoxemia in rats with diet-induced obesity [7].

Despite the growing efforts to study the mechanisms by which apple consumption can improve health, the effect of apple supplementation on health and the gut microbiota is still not well understood. For example, we know that certain enterotypes are strongly associated with long-term dietary patterns, particularly protein and animal fat versus carbohydrates [2], but whether the effect of apple supplementation is dependent on the composition of the diet has not been investigated. The aim of this study was to investigate the effect of apple supplementation on the fecal microbiota of rats exposed to a high-fat and a low-fat diet, and to determine how microbiota changes can be related to changes in relevant gut biomarkers and metabolites.

## Materials and methods

### Study design

This study was reviewed and approved by the Institutional Animal Care and Use Committee at Washington State University (IACUC # 04436–001). Six weeks old male Sprague-Dawley rats (Harlan Laboratories, Kent, WA, USA, body weight: 170–210 g) were housed in a controlled environment with a 12 h light/dark cycle. Food and water were supplied *ad libitum*. Food intake was recorded every 2–3 days and body weight was recorded weekly. Animals were divided into four groups: high fat (HF) group (n = 14), HF supplemented with 5% freeze dried apple (HFA, n = 14); low fat (LF) group (n = 5) and LF supplemented with 5% apple (LFA, n = 6). Formulation of the HF diet was based on D12492 with modifications (Research diet, 60% kcal from fat, New Brunswick, NJ, USA), and LF was based on D12450B with modifications (Research diet, 10% kcal from fat, New Brunswick, NJ, USA) (Table 1). Granny Smith apples grown in Washington State were bought from a local Walmart in Pullman, Washington, USA, and treated with 90°C for 5 min [8] to condition the apples for food preparation. This treatment likely destroyed most apple-associated microorganisms and this is important considering that apples harbor their own microbial populations [9] and that this microbiota may exert additional effects *in vivo*. Freeze dried Granny Smith apples (27.57 ± 0.16% moisture) contained 0.98 ± 0.12 mg gallic acid equivalents (GAE)/g dry weight (DW) as total extractable phenolics, 4.10 ± 0.46 mg proanthocyanidins/g DW as non-extractable phenolics, 25.9 ± 1.14% DW of dietary fiber (16.0 ± 1.14% insoluble fiber and 9.9 ± 0.14% soluble fiber). Sucrose was used in replacement of apple to maintain isocaloric contents between supplemented and non-supplemented diets (more information about the source of the apples and diet preparation is available in S1 Supporting Information). The diets were offered for a period of six weeks. Body mass index (BMI, kg/m^2^) were calculated as body weight (kg)/height squared (without the tail, m^2^). Adiposity percentage was determined by the sum of fat tissues (epididymal fat and abdominal fat divided by body weight multiplied by 100).

**Table 1:** Composition of diets used in this study.

Treatment	HF		HFA		LF		LFA	
Macro ingredient	Kcal%		Kcal%		Kcal%		Kcal%	
Protein	20		20		20		20	
Carbohydrate	20		20		70		70	
Fat	60		60		10		10	
Total	100		100		100		100	
Ingredient	gm	kcal	gm	kcal	gm	kcal	gm	kcal
Casein	133	533	133	533	133	533	133	533
L-Cystine	2	8	2	8	2	8	2	8
Corn starch	0	0	0	0	337	1350	337	1350
Maltodextrin	83	333	83	333	83	333	83	333
Sucrose	46	183	0	0	46	183	0	0
Soybean oil	17	150	17	150	17	150	17	150
Cellulose	33	0	33	0	33	0	33	0
Mineral mix^a^	7	0	7	0	7	0	7	0
Vitamin mix^b^	7	27	7	27	7	27	7	27
Choline bitartrate	1	0	1	0	1	0	1	0
t-Butylhydroquinone	0	0	0	0	0	0	0	0
Lard	163	1470	163	1470	13	120	13	120
Apple	0	0	46	183	0	0	46	183
Water	480	0	480	0	480	0	480	0
Agar	20	0	20	0	20	0	20	0
Total	993	2705	993	2705	1180	2705	1180	2705

HF: high-fat diet; HFA: apple-supplemented HF diet; LF: low-fat diet; LFA: apple-supplemented LF diet.

^**a**^**AIN-93G-MX** supplied by Dyets Inc. (Bethlehem, PA), containing (g/kg): Calcium Carbonate (357), Potassium Phosphate, monobasic (196), Potassium Citrate .H20 (70.78), Sodium Chloride (74), Potassium Sulfate (46.6), Magnesium Oxide (24), Ferric Citrate, U.S.P. (6.06), Zinc Carbonate (1.65), Manganous Carbonate (0.63), Cupric Carbonate (0.3), Potassium Iodate (0.01), Sodium Selenate (0.01025), Ammonium Paramolybdate .4H20 (0.00795), Sodium Metasilicate .9H20 (1.45), Chromium Potassium Sulfate .12H20 (0.275), Lithium Chloride (0.0174), Boric Acid (0.0815), Sodium Fluoride (0.0635), Nickel Carbonate (0.0318), Ammonium Vanadate (0.0066), Sucrose, finely powdered (221.026).

^**b**^**AIN-93G Vitamin Mix** supplied by Dyets Inc. (Bethlehem, PA), containing (g/kg): Niacin (3), Calcium Pantothenate (1.6), Pyridoxine HCl (0.7), Thiamine HCl (0.6), Riboflavin (0.6), Folic Acid (0.2), Biotin (0.02), Vitamin E Acetate (500 IU/g) (15), Vitamin B12 (0.1%) (2.5), Vitamin A Palmitate (500 000 IU/g) (0.8), Vitamin D3 (400 000 IU/g) (0.25), Vitamin K1/Dextrose Mix (10 mg/g) (7.5), Sucrose (967.23). The election of an agar-based diet allowed fulfilling the nutrients and part of the water requirement of mice [10].

### Biochemical and inflammatory biomarkers in blood and plasma

Euthanasia was performed by gradual exposure to inhalation with CO_2_ until the animal became unconscious. Blood was collected from the unconscious animal by cardiac puncture (100–500 μL) into a tube containing 10 μL of ethylenediaminetetraacetic acid (EDTA) (Sommerville, NJ USA) and centrifuged at 5000 g at 4°C for 10 min to separate plasma from erythrocytes. Thereafter animals were killed by cervical dislocation. Plasma was aliquoted and stored in -80°C for future use. The concentrations of lipopolysaccharides (LPS) were estimated in plasma using an endotoxin analysis (Endpoint Chromogenic LAL Assay) following the manufacturer’s protocol (Lonza Walkersville, Inc., Walkersville, MD, USA). Plasma glucose levels were determined by glucose enzymatic test using Amplex Red Enzyme Assay (Life Technologies, Carlsbad, CA); triglycerides, HDL-cholesterol, and total cholesterol concentrations were quantified using commercial kits according to the manufacturer’s protocols (Wako Diagnostics, Richmond, VA, USA); lipid peroxidation were measured using the thiobarbituric acid reactive substance (TBARS) assay (Cayman Chemical Co. Ann Arbor, MI), according to the manufacturer’s protocol. The level of lipid peroxidation was expressed as μM malondialdehyde (MDA). The mutiplex Rat Adipokine kit (Millipore, Billerica, MA) was used to quantify interleukin 6 (IL-6), interleukin-1beta (IL-1β), tumor necrosis factor alpha (TNF-α), plasminogen activator inhibitor-1 (PAI-1), leptin, insulin and monocyte chemoattractant protein-1 (MCP-1) using the Mutiplex Rat Adipokine kit (Millipore, Billerica, MA). Data was analyzed using Luminex xPonent3.0 software (Austin, TX, USA).

### Analysis of fecal microbiota

#### DNA extraction and 16S sequencing

Fecal samples were obtained at the end of the study (i.e. after 6 weeks of dietary supplementation) and used for DNA extraction, purification and later PCR amplification and sequencing as described elsewhere [11]. The primers F515 (5´–GTGCCAGCMGCCGCGGTAA–3´) and R806 (5’–GGACTACHVGGGTWTCTAAT–3’) were used to amplify the V4 semi-variable region of the 16S rRNA gene. Sequencing was performed using a MiSeq instrument following the manufacturer instructions (Molecular Research LP, Shallowater, TX, USA) as described elsewhere [11].

#### Bioinformatics

The Quantitative Insights in Microbial Ecology (QIIME) bioinformatics pipeline [12] v1.8 was used for performing microbiome analysis. Operational Taxonomic Units (OTUs) were selected using two approaches. First, using an open approach as described by Rideout et al. [13]. Before performing taxonomic and diversity analyses, we removed very low abundant OTUs (i.e. OTUs with <0.005% sequences) to increase the sensitivity of detecting true phylogroups as suggested by Navas-Molina et al. [14]. Second, using a closed approach for later use in predicting metagenome content (see Prediction of functional metagenome). The GreenGenes [15] version 13_5 OTUs and taxonomy at 97% similarity were used for OTU picking. All data is freely available at the Sequence Read Archive of the NCBI (Bioproject: PRJNA504388).

### Prediction of functional metagenome

Phylogenetic Investigation of Communities by Reconstruction of Unobserved States (PICRUSt) [16] was used to predict the metagenome content using the OTU table file from the closed reference approach described above.

### qPCR analysis

Quantitative real-time PCR (qPCR) analysis was used to quantify the abundance of a subset of bacterial groups (S1 Supporting Information) in an effort to expand the sequencing results. qPCR data is expressed as log amount of DNA (picograms of amplified DNA) for each bacterial group per 10 ng of total DNA.

### Biomarkers and metabolites in fecal samples and colonic mucosal cells

#### Short-chain fatty acids (SCFAs) in fecal samples

Several SCFAs were measured in fecal samples using high-performance liquid chromatography (HPLC) as reported elsewhere [11]. Briefly, samples were analyzed using a HPLC-PDA system with an Aminex HPX-87H strong cation-exchange resin column and fitted with an ion exchange microguard refill cartridge. Sodium butyrate, acetic acid, oxalic acid and succinic acid were identified and quantified by comparing retention time and UV-Visible spectral data to standards [11].

#### mRNA analysis of colonic mucosal cells

Colonic mucosal cells scrapped from terminal colon were collected from all rats and mechanically pulverized in liquid nitrogen. RNA was extracted from these samples using TRIzol LS Reagent (Life technologies, Carlsbad, CA, USA) according to the manufacturer’s protocol. Purified mRNA was used to measure several biomarkers of gut health (e.g. interleukins) with primers presented in S1 Supporting Information.

### Proteomics and metabolomics analysis

Proteomic analyses of fecal samples and colonic mucosal cells were performed using mass spectrometry at the Tissue Imaging and Proteomics Laboratory. Fecal metabolomic analysis was performed using gas chromatography time-of-flight mass spectrometry at the Laboratory for Cellular Metabolism and Engineering at WSU as detailed in S1 Supporting Information.

### Statistical analyses

In this study we used the linear discriminant analysis (LDA) effect size (LEfSe) method [17], which uses the non-parametric Kruskal-Wallis sum-rank test to detect features (in this case bacterial taxonomy groups) with significant differential abundance with regards to a class of interest (e.g. diet in this study). Please note that in LEfSe the idea is that the significant biomarkers are ranked based on the effect size ("the magnitude of the variation") rather than on the statistical significance. We also compared the frequencies of OTUs using the group_significance.py script in QIIME (the Kruskal-Wallis test was used to compare across all 4 treatment groups). The Unique Fraction metric (UniFrac) was used for comparing microbial communities [18,19]. PICRUSt results were visualized and analyzed using STAMP [20]. Statistical analyses of proteomics and metabolomics data were performed on normalized data using MetaboAnalyst 3.0 [21]. These analyses were only performed on samples from the HF group because of the higher number of samples. Partial least squares-discriminant analysis (PLS-DA) and hierarchical clustering based on Euclidean distance with only significant proteins or metabolites from the non-parametric t-test, Wilcoxon-sum test, considering a false discovery rate cutoff 0.05 were also performed (S1 Supporting Information).

## Results and discussion

### Body weight gain over time

**Fig 1:**
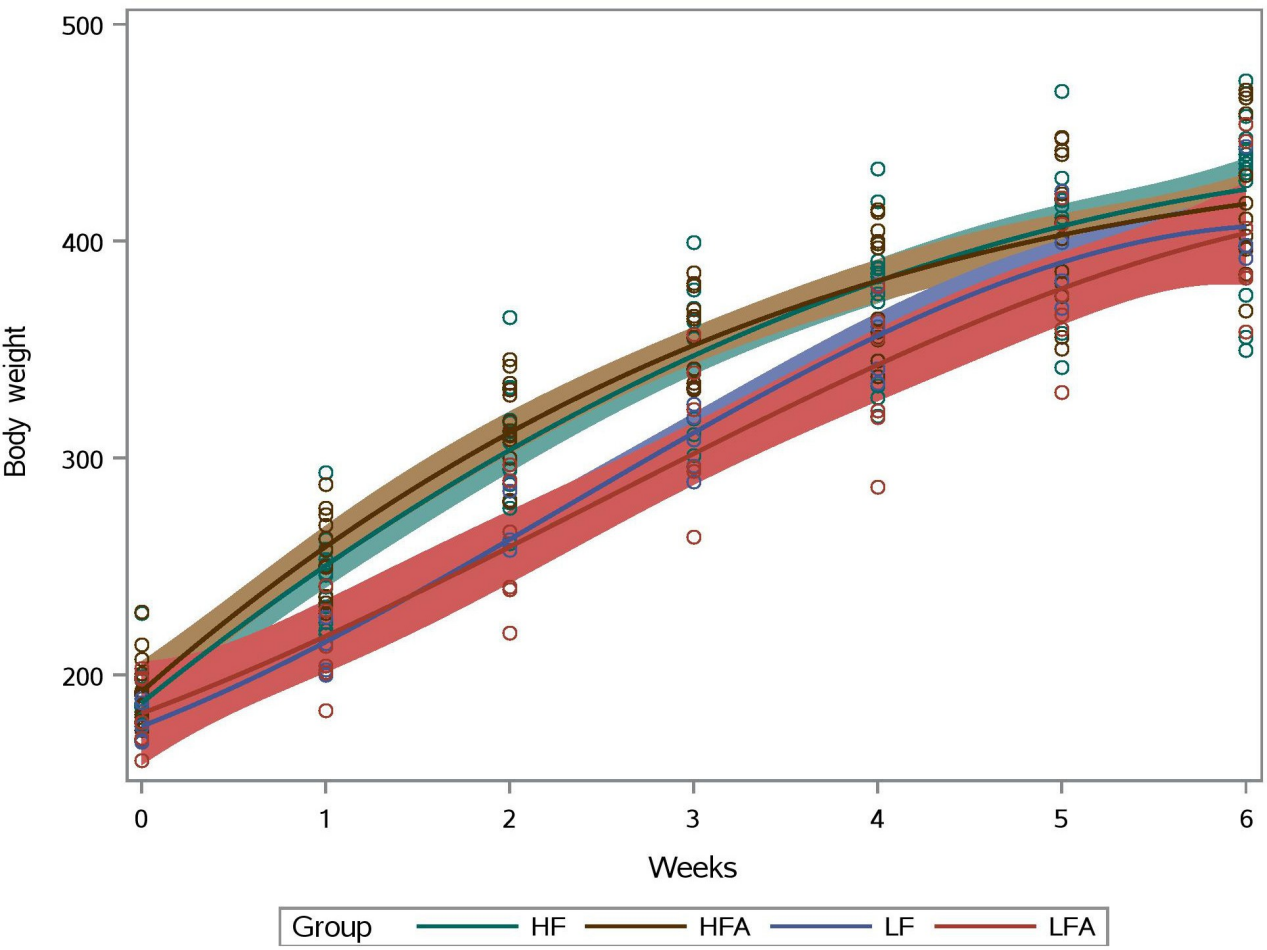
Body weight (in grams, y axis) gain over time. HF: high-fat group; HFA: supplemented HF group; LF: low-fat group; LFA: supplemented LF group. The area surrounding the slopes represent 95% confidence limits.

One animal in the supplemented HF group died for unknown reasons. Interesting relationships between body weight gain and dietary modification have been shown by our research group, for example using supplementation with quinoa [11]. In this study, all subjects had a similar body weight gain over time but there was a clear separation between HF and LF groups between week 1 and 4 (Fig 1). However, there was no difference in final body weight (week 6) between supplemented and control subjects or between the HF and the LF group. Food intake over time was similar among all treatment groups with the exception of the LF group that showed a wider variation in food intake over time (S1 Supporting Information). Consistent with these results, BMI and organ weights were similar in either group (HF or LF) with and without apple supplementation.

### Biochemical and inflammatory biomarkers in blood and plasma

LPS are large endotoxins found in gram-negative bacteria that can elicit a strong immune response. There was a statistically lower concentration of LPS in blood of HF apple-supplemented subjects compared to controls in the HF group (median = 0.85 and 2.3 EU/mL, respectively) (*P* = 0.002, Mann-Whitney test). This finding may be particularly relevant in the context of blood-brain barrier [22]. No difference was found between the LF groups with and without supplementation groups whose LPS levels were lower than HF. The lower LPS levels in HFA group is relevant and strongly suggests that apple consumption can be an effective approach to protect from inflammatory reactions and systemic exposure to intestinal bacteria components. This mechanism seems to be modulated by apple fiber and polyphenols that alter colon microbiota and the absorption of bacterial products from the intestinal lumen. Previous studies with cranberry and wine polyphenols reported decreased plasma LPS levels in diet-induced obesity C57BL/6J mice [23] and obese adult men [24], respectively.

In the HF group, results showed that apple supplementation was associated with lower glucose levels (6.3 ± 0.7 vs 7.0 ± 1.0 mg/dL in the HFA and HF groups, respectively), plasma LDL oxidation (143.5 ± 3.3 vs 149.7 ± 6.8 μM MDA), leptin (1760 ± 332 vs 2400 ± 317 pg/mL); insulin (2180 ± 1670 vs 2979 ± 547 pg/mL); and MCP-1 (197 ± 27 vs 284.2 ± 41 pg/mL). Apple supplementation did not influence these biomarkers in LF groups. Overall, these results indicate that apple intake may lessen plasma biomarkers associated with disorders tied to obesity.

### Analysis of fecal microbiota

The effect of apple intake on the gut microbiota has not been well studied. Shinohara et al. [25] investigated the effect of consuming two apples a day for two weeks on the fecal microbiota of eight healthy volunteers and showed increased abundances of *Bifidobacterium* and a tendency for increased abundances of *Lactobacillus*, *Streptococcus* and *Enterococcus*. Unfortunately, the authors only used culture techniques which are known to lack enough resolution to fully describe microbial communities. Other studies have analyzed the effect of apple consumption *in vitro* [26] and *in vivo* but for a few selected groups only [5]. This topic is relevant for human health, for instance growing evidence shows that the gut microbiota is a core consideration for the effect of apple consumption in cardiovascular diseases [27].

**Fig 2:**
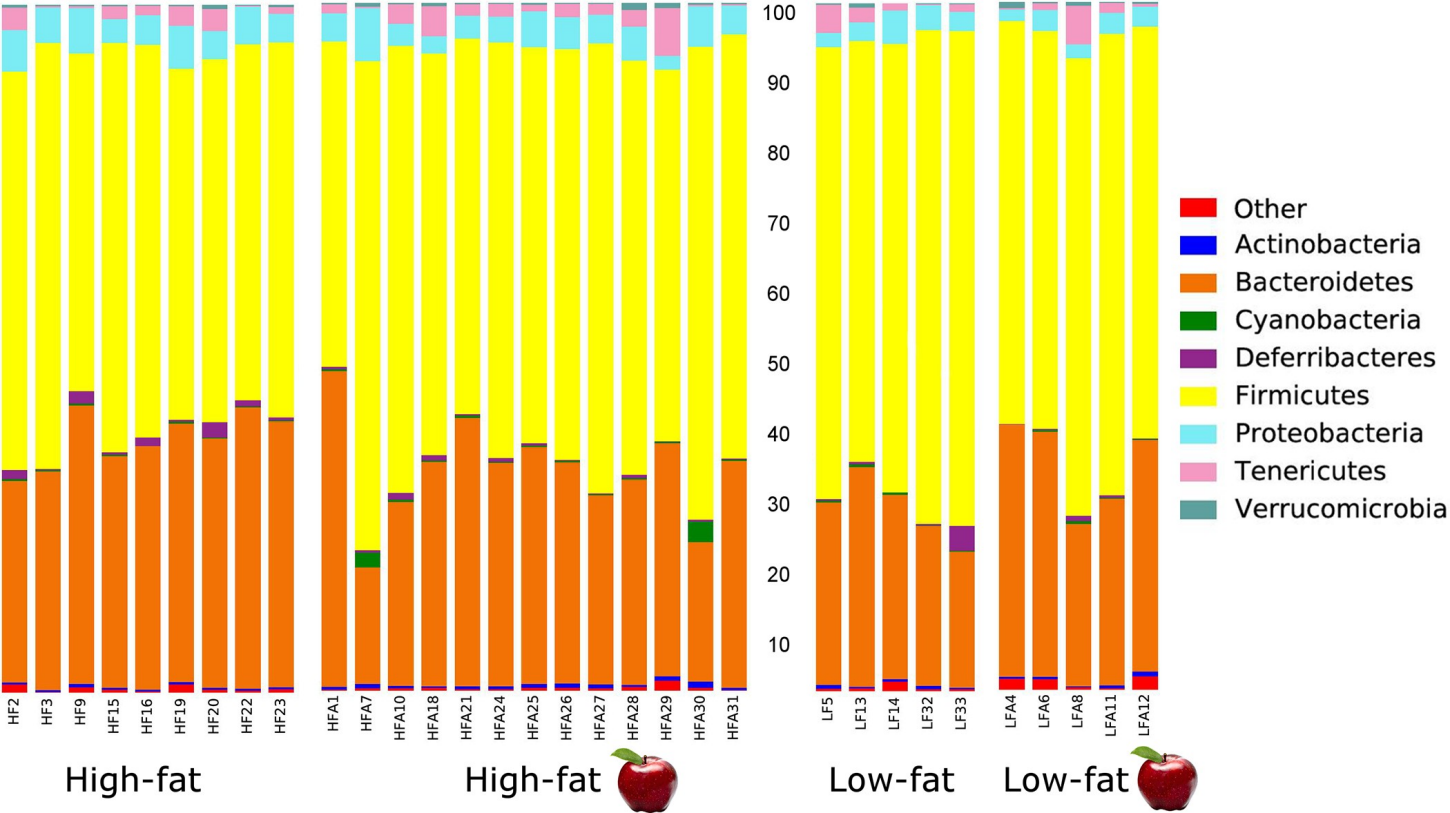
Stacked bar chart showing relative proportions (percentages of 16S sequences) of bacterial microbiota at the phylum level.

**Fig 3:**
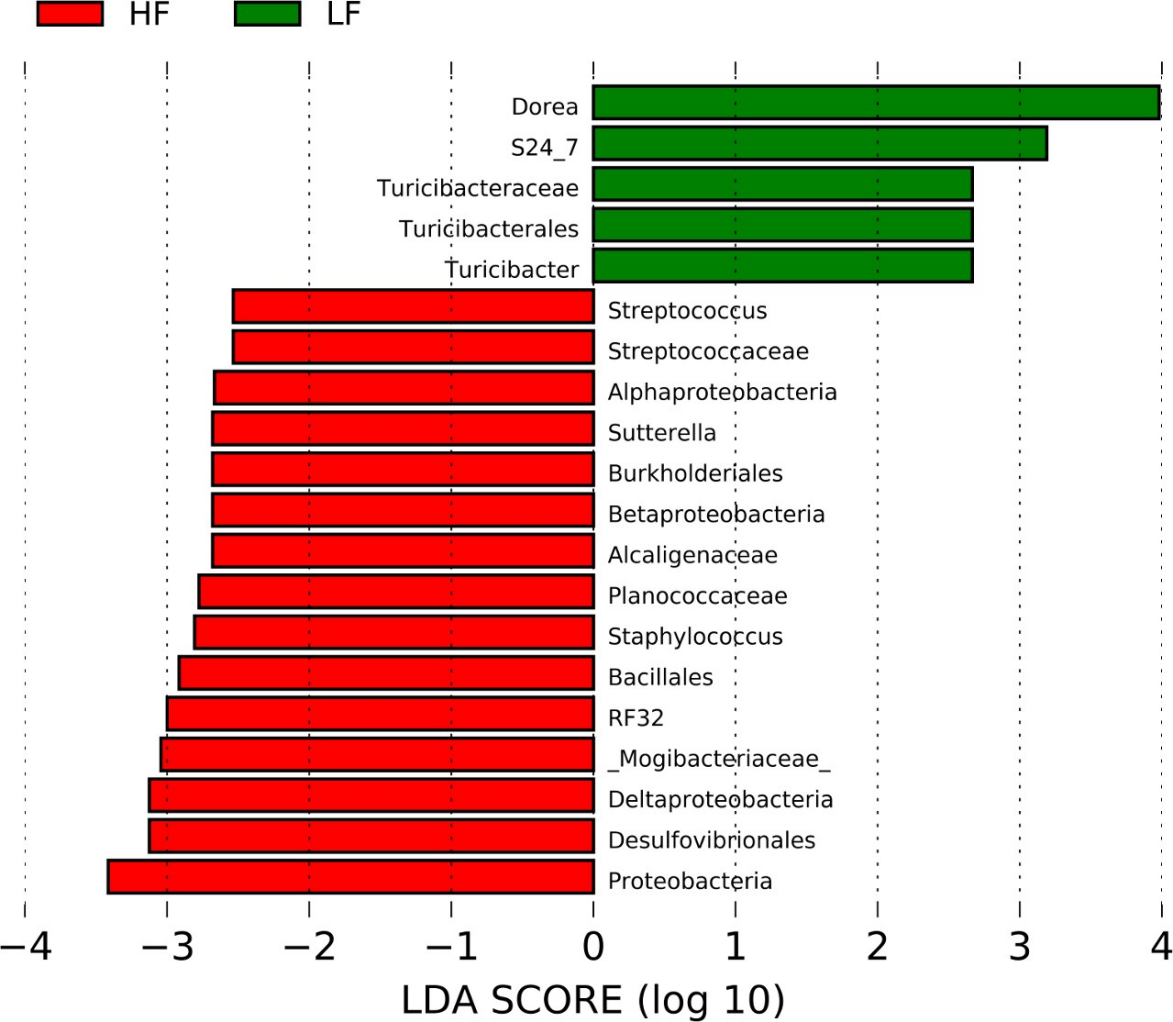
LEfSe results. This figure shows the bacterial groups that were different in abundance between the high-fat (HF) and the low-fat (LF) groups.

Total genomic DNA was successfully isolated from all fecal samples but sequencing was performed only in a subset of samples from the HF group (9/14) and the LFA group (5/6) because of unsatisfactory DNA quality. The open OTU picking approach described above showed that the microbiota of all samples (n = 32) was composed by 69,010 different OTUs using the original OTU table. After removal of low abundant OTUs, a total of 1,339 OTUs were detected using the same open approach (min: 38,099, max: 156,470 sequences per sample). Using a rarefaction depth of 38,000 sequences, the microbiota was dominated by members of Firmicutes, Bacteroidetes, Proteobacteria and others (Fig 2). LEfSe revealed differences in relative proportions of multiple taxa between the HF and the LF groups (Fig 3), a phenomenon that, although may seem congruent with our current knowledge, is interesting because at the time of fecal sampling the animals had similar body weights (Fig 1). Interestingly, there was a strong negative linear relationship between the relative abundance (i.e. proportions of 16S) of Firmicutes and Bacteroidetes in the HF group (*P* < 0.0001, R^2^ = 0.89) and this was also true when analyzing all samples, including the LF group. No other significant correlation was found among the different phyla. However, the correlation was positive when using the real number of sequences instead of the proportion of sequences (*P* = 0.0002, R^2^ = 0.37). Although this subject may seem meaningless, it is important because of the emphasis in the literature on the so-called Firmicutes-Bacteroidetes ratio (see below).

**Fig 4:**
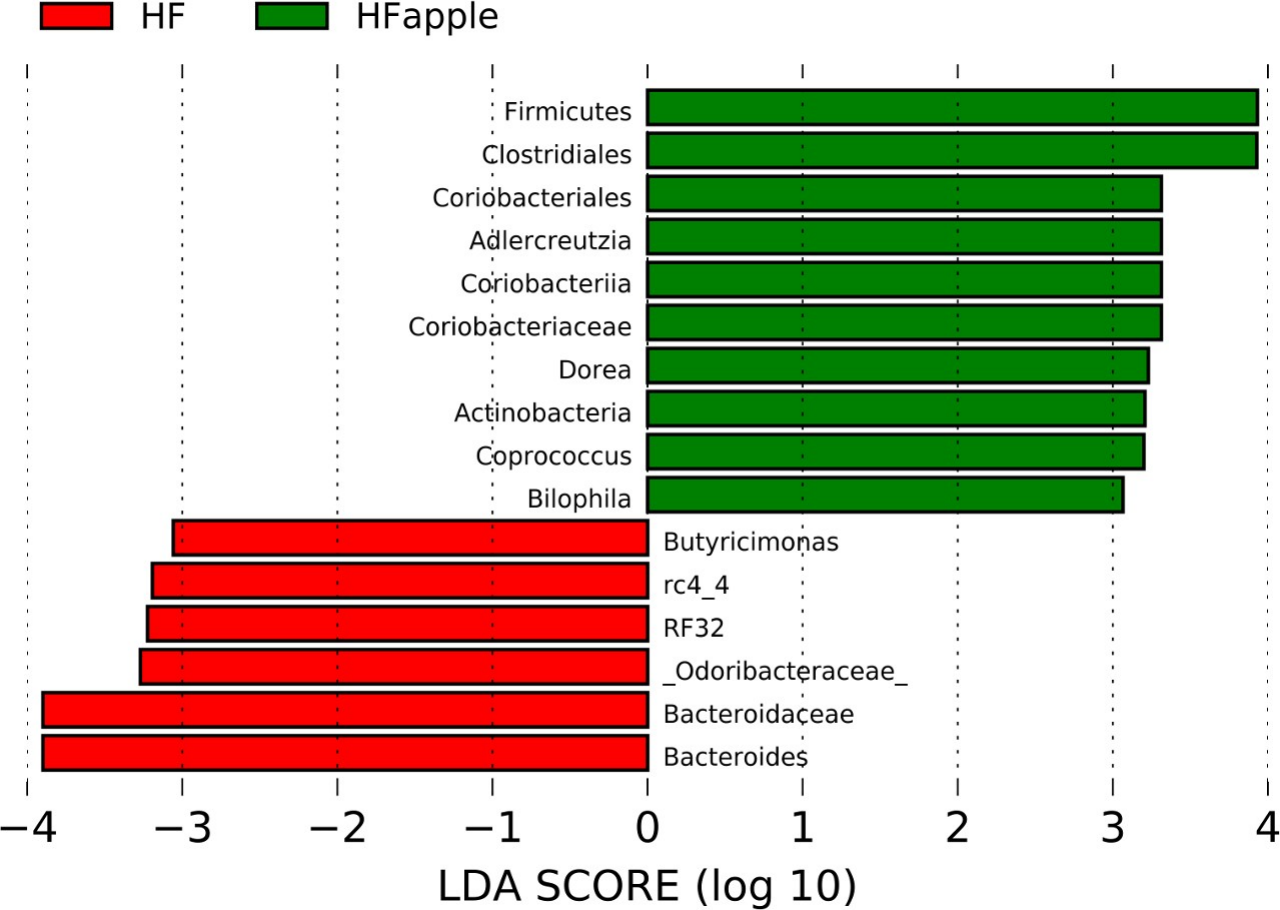
LEfSe results. This figure shows the bacterial groups that were different in abundance between the high-fat (HF) controls and the HF supplemented (HFapple) group.

LEfSe analysis revealed an increased abundance of *Blautia* in apple-supplemented rats (~1%) compared to controls in the LF group (~0.5%); other than this, there were not any relevant differences to be mentioned or discussed with regards to the LF group. The difference in *Blautia* in the LF group is interesting though because it displays host-specificity and host-preference patterns believed to be driven by host physiology more than dietary habits [28]. Within the HF group, the ratio Firmicutes:Bacteroidetes (shown to be higher in obese mice compared to lean counterparts in some studies, see Ley et al. [29]) was higher in apple-supplemented rats (median: 2.1) compared to controls (median: 1.6) and this difference almost reached significance (*P* = 0.06). At lower taxonomic levels, apple-supplemented rats had higher abundance of an unknown group of Clostridiales (*P* = 0.01) and lower abundance of the family Bacteroidaceae (*P* = 0.02) (Fig 4). The ratio Clostridiales:Bacteroidaceae was higher in apple-supplemented rats (median: 1.0) compared to controls (median: 0.6), and this difference was statistically stronger (*P* = 0.003) compared to the difference in Firmicutes:Bacteroidetes ratios. Overall, this supports the results at the phylum level and suggest that apple supplementation increases some members of Clostridiales and lowers some members of Bacteroidaceae in the presence of HF diet, in contrast to the results by Masumoto et al. [4] in mice. Please note that more Clostridiales and less Bacteroidaceae is considered by some authors to be suboptimal for gut health but this thought is unfounded (see “Studies in humans” in Delzenne and Cani, [30]).

In the HF group, the relative abundance of important fiber (the S24-7 group) or mucin (*Akkermansia*) degraders did not show difference between apple-supplemented and controls accordingly to LEfSe analysis. Note that in the case of *Akkermansia* the results at the mucosal level likely would have resulted in different results. Similarly to other studies [26], the abundance of Actinobacteria was increased in apple-supplemented rats (~0.45%) compared to controls (~0.29%, *P* = 0.025) but in this study this change was not related to *Bifidobacterium* (~0.3% in all rats regardless of treatment group, *P* = 0.83). Another group that is very important for gut health is Lachnospiraceae [31], which in this study showed higher abundance in HF apple-supplemented rats (9.9%) compared to controls (7.7%, *P* = 0.04). *Odoribacter*, which has been shown to be increased in hyperlipidimic laboratory animals [32], was lower in apple-supplemented (1.7%) compared to control (3.9%, *P* = 0.04), a phenomenon that can also be considered positive for health. Other groups that showed a significant difference between HF apple-supplemented and controls are shown in Fig 4. Again, please note that these differences are interesting since the samples were taken at week six when all animals had similar body weights (see Fig 1).

### Analysis at the OTU level

The analysis of relative proportions of 16S sequences assigned as whole groups or taxa often misses potential differences at the OTU level, a topic of great relevance in microbial ecology [33]. In this study, the comparison of OTU frequencies revealed that the changes in individual OTUs may not mimic the changes in whole taxa (S1 Supporting Information). Moreover, this additional analysis revealed the possibility that some OTUs may increase in abundance upon apple consumption in the LF group (e.g. unclassified members of Clostridiales and Ruminococcaceae), a phenomenon that was not noticed in the analysis of relative proportions of phylotypes.

### Diversity analyses

**Fig 5:**
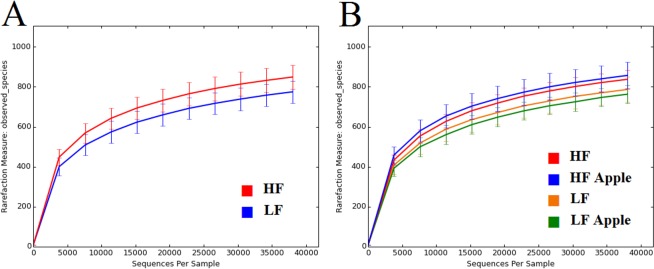
Number of OTUs (observed species, y axis) as a function of sequences per sample (x axis). A: comparison between the high-fat (HF) and the low-fat (LF) group. B: comparison among all groups with and without apple supplementation.

The number of observed “species” (i.e. OTUs at 97% similarity) revealed an interesting pattern of variation among the four treatment groups. First, overall HF rats had a relatively higher number of OTU types (940, range: 802–1090) compared to LF rats (865, range: 778–980) (*P* = 0.0097, Fig 5A) at the same rarefaction depth. Interestingly, apple supplementation was associated with relatively higher and lower numbers of OTU types in the HF and the LF groups, respectively (Fig 5B), although this difference did not reach significance. Shannon diversity indexes, which considers both the estimates of richness (i.e. number of different species) and evenness (i.e. how close in numbers each species is), were higher (*P* = 0.03) in apple-supplemented rats within the HF group (Table 2).

**Table 2:** Median (minimum-maximum) values for alpha metrics.

Metric	HF (n = 9)	HF apple (n = 13)	*P* value
Shannon	6.9 (6.6–7.2)	7.1 (6.6–7.6)	0.03
Observed species	937 (844–1090)	942 (802–1039)	0.63
PD whole tree	105 (100–114)	105 (97–111)	0.69
Chao1	1095 (969–1196)	1056 (929–1129)	0.12
Metric	LF (n = 5)	LF apple (n = 5)	*P* value
Shannon	6.9 (6.2–7.3)	6.9 (6.1–7.1)	0.22
Observed species	912 (778–980)	838 (814–886)	0.22
PD whole tree	103 (96–107)	99 (98–102)	0.15
Chao1	1040 (949–1044)	986 (921–1009)	0.06

HF: high-fat group; HF apple: supplemented HF group; LF: low-fat group; LF apple: supplemented LF group.

**Fig 6:**
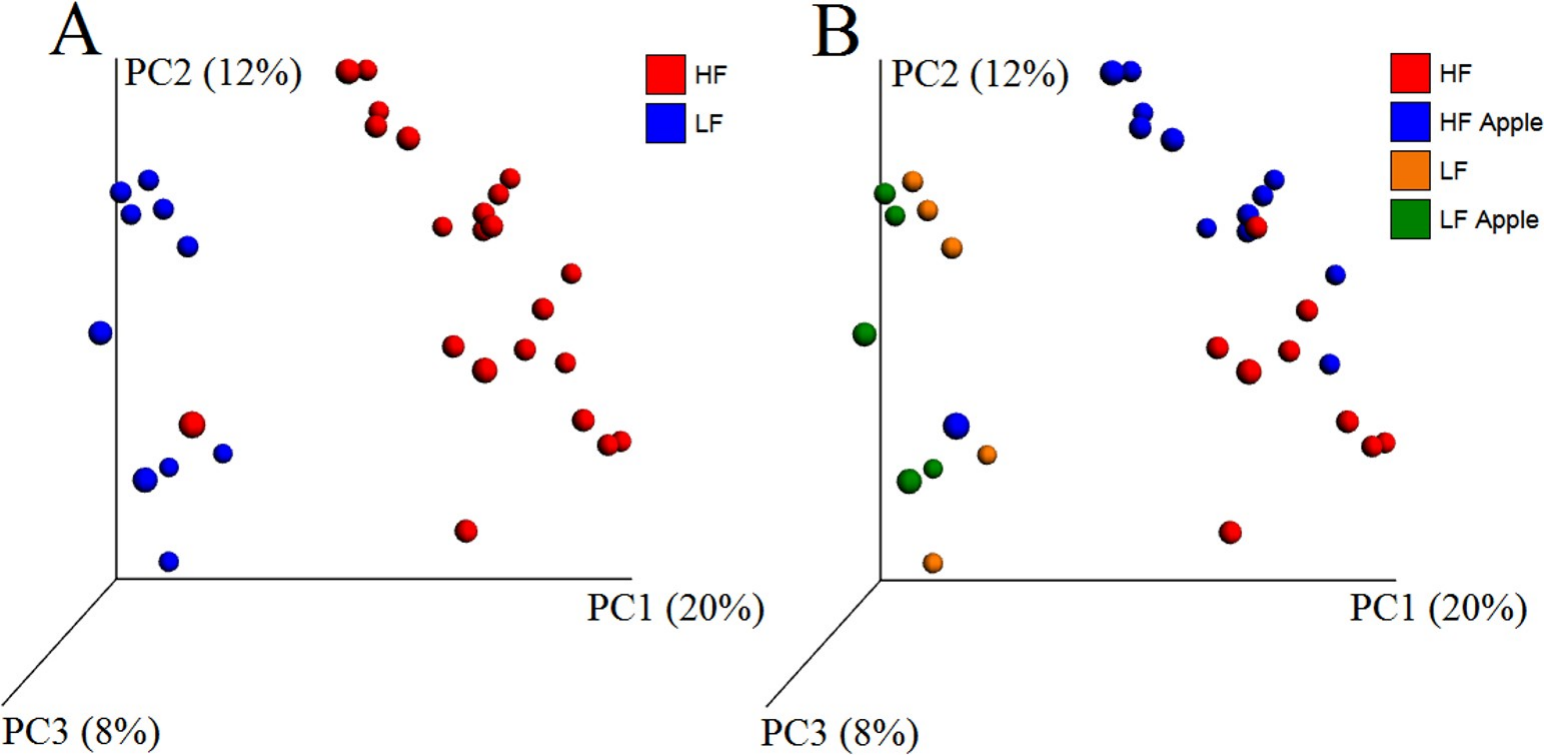
PCoA plots using unweighted UniFrac distances. A: comparison between the high-fat (HF) and the low-fat (LF) group. B: comparison among all groups with and without apple supplementation.

The analysis of unweighted UniFrac distances revealed a clear and strong separation of bacterial communities based on diet (HF vs LF) and less strongly on Treatment (HF and LF with and without apple supplementation) (Fig 6 and Table 3). The comparison of communities between the samples with and without apple supplementation (regardless of diet, all samples included) did not yield any significance (R = 0.07 ANOSIM test, Table 3), thus further supporting that diet (HF vs LF) was the main driver of differences. The results from weighted UniFrac distances did not show strong clustering of samples based on Diet or Treatment as revealed by very low R values (<0.2) in ANOSIM tests (Table 3), thus implying that the clustering of samples was not so much due to differences in taxon abundance but rather to what type of organisms were able to thrive in a particular environment (e.g. an environment with high fat dietary content). These results were confirmed using all OTUs (i.e. original unfiltered OTU table) and OTUs from the HF rats only. Importantly, we performed beta diversity analysis of several subgroups of sample sizes within the HF group using UniFrac distances and the differences in bacterial communities were consistent, thus implying that the lack of significant clustering between supplemented and non-supplemented rats in the LF group was likely not due to the lower number of samples.

**Table 3:** *P* and R values from clustering analysis using the Adonis and ANOSIM tests using unweighted and weighted UniFrac distances.

	Unweighted UniFrac		
	Apple supplementation (with vs without)^a^	Diet (HF vs LF)^b^	Treatment (HF and LF with and without supplementation)^c^
Adonis	*P* = 0.071	*P* = 0.001	*P* < 0.001
ANOSIM	*P* = 0.071 (R = 0.07)	*P* = 0.001 (R = 0.69)	*P* = 0.001 (R = 0.54)
	Weighted UniFrac		
Adonis	*P* = 0.072	*P* = 0.011	*P* = 0.005
ANOSIM	*P* = 0.129 (R = 0.05)	*P* = 0.028 (R = 0.19)	*P* = 0.009 (R = 0.18)

Results are shown from the comparison of all samples with (n = 18) and without (n = 14) apple supplementation, regardless of diet^a^; from the comparison between the high-fat (HF, n = 22) and the low-fat (LF, n = 10) group, regardless of apple supplementation^b^; and from the comparison of all four treatment groups^c^. These results are consistent with the clustering of samples in Fig 6.

### Predicted metabolic profile using PICRUSt

A total of 2,095 OTUs were detected in all samples using the closed OTU picking approach and used for metagenome predictions using PICRUSt. This approach yielded a total of 242 features; however, when adjusted with Bonferroni correction in STAMP, there was no significant difference in any predicted feature among the four treatment categories (HF and LF diets with and without apple supplementation, lowest adjusted *P* ≥ 0.79, Kruskal-Wallis test). Interestingly, this lack of significant differences was also true when analyzing OTUs from the HF group only (lowest adjusted *P* ≥ 0.97). This implies that the differences in the abundance of taxa, whole communities, and diversity were not sufficient to produce a different predicted metagenomic profile. Interestingly, the analysis of OTUs from Bacteroidaceae only (either from all groups or from the HF group only) and other abundant groups also did not reveal any treatment effect. This is interesting because other studies from our research group have shown divergent results when analyzing separate taxa (i.e. using filtered OTU table) as opposed to all taxa (i.e. full OTU table) [34].

### qPCR analyses

We performed qPCR analyses in an effort to expand the results offered by deep sequencing (e.g. more Clostridiales, less Bacteroidetes in supplemented rats within the HF group). Unfortunately, we did not find evidence to suggest that apple consumption changes the abundance of Firmicutes (*P* = 0.46) or Bacteroidetes (*P* = 0.94) in the HF group. Interestingly, apple supplementation was associated with a relatively lower abundance of fecal *Turicibacter* (*P* = 0.048) but this group was very low (~0.03% on average) and similar among all samples in sequencing results. Moreover, and in line with the results using the real number of sequences, there was a strong positive linear relationship between the abundance of Firmicutes and Bacteroidetes in the HF group (*P* < 0.0001, R^2^ = 0.68), and this was also true when analyzing all samples and for the relationship between Ruminococcaceae and *B*. *fragilis*. The discrepancy between qPCR and sequencing results is due to various factors such as primer specificity and nature of output results (e.g. compositional data vs true abundances).

### Biomarkers and metabolites

The reasons behind any beneficial effect of apple supplementation on health and the gut microbiota are likely to be multifactorial. For example, pectin binds to cholesterol in the gut and slows glucose absorption by trapping carbohydrates [35]; therefore, the effect on the distal gut microbiota could have been indirect (i.e. a result of different chemical composition of the bolus reaching the lower gut). Moreover, phenolics and other bioactive compounds are transformed throughout the digestive tract thus reaching the lower gut in a modified form [36] and the microbial biochemistry inside the gut is very complex [37]; therefore the increase in the abundance of one bacterial group could be simply the result of the decrease in other groups. For these and other reasons, it is also relevant to look at biomarkers and metabolites to better understand any potentially beneficial effect of apple consumption.

### SCFAs in feces

There was no difference in the concentration of any SCFAs in feces (sodium butyrate, sodium propionate, and acetic acid) when compared supplemented vs control subjects for either the HF or the LF group (S1 Supporting Information). There was also no difference when comparing baseline values from the HF and LF group. Please note that microbial formation of SCFA is regulated by many host and dietary factors [38], therefore the analysis of all SCFAs (without discerning the identity of the producer) is hampered by the fact that individual groups of microbes can change their own biochemical behavior with or without affecting the overall biochemical pool of compounds.

### mRNA analysis in colonic mucosal cells

Results from mRNA analysis showed no difference in mRNA levels between LF and LFA groups for all the assessed genes (not shown). Similarly, mRNA levels of results were found between HF and HFA with the exception of TGF-β1, which was significantly upregulated in HFA group (*P* = 0.0157, Mann-Whitney test) (S1 Supporting Information). TGF-β1 is an anti-inflammatory cytokine, recognized as a key regulator of immunological homeostasis and inflammatory responses, acts as a negative regulator of mucosal inflammation. *In vitro* and *in vivo* studies showed that TGF-β1 activation leads to decreased production of inflammatory cytokines in both colitic mice and inflammatory bowel disease patients and attenuates clinical activity in Crohn's disease patients (reviewed in Sedda et al. [39]). The effect of apple supplementation on increasing the gene expression of TGF- β1 might be attributed to the fiber content in apple. Dietary fiber has shown to improve intestinal function, in part mediated by changes in microbiota composition (increased *Bifidobacterium* counts in colon) and to increase expression of TGF- β1 [40]. These results have important implications and suggest a role of apple in protecting from intestinal dysfunction and injury caused by high fat diet.

### Proteomics and metabolomics

Analysis of proteomics and metabolomics data was performed only considering the results from HF and HFA groups because of the higher number of subjects in these experimental groups compared to the LF and LFA groups. Non-parametric test (Wilcoxon rank-sum test) allowed the identification of 127 proteins that were differentially expressed in feces of HF and HFA (S1 Supporting Information). These proteins were mostly related to the transcription elongation factor GreA and were predicted to be produced by multiple members of the microbiota, particularly Lachnospiraceae and other members of the order Clostridiales within the Firmicutes (S1 Supporting Information). The PLS-DA in two components discriminated HF from HFA groups and explained 31.8% of the total variance (S1 Supporting Information), while PLS-DA in three components discriminated HF from HFA groups and explained 40% of the total variance (S1 Supporting Information). Furthermore, hierarchical cluster analysis (S1 Supporting Information), illustrates that these proteins are in relative higher abundance in 7 out of 11 subjects of HFA group and in 1 out of 12 subjects of HF group. Considering that most of these proteins were produced by Lachnospiraceae and other members of the order Clostridiales, these results are consistent with our sequencing results in apple-supplemented rats within the HF group.

**Fig 7:**
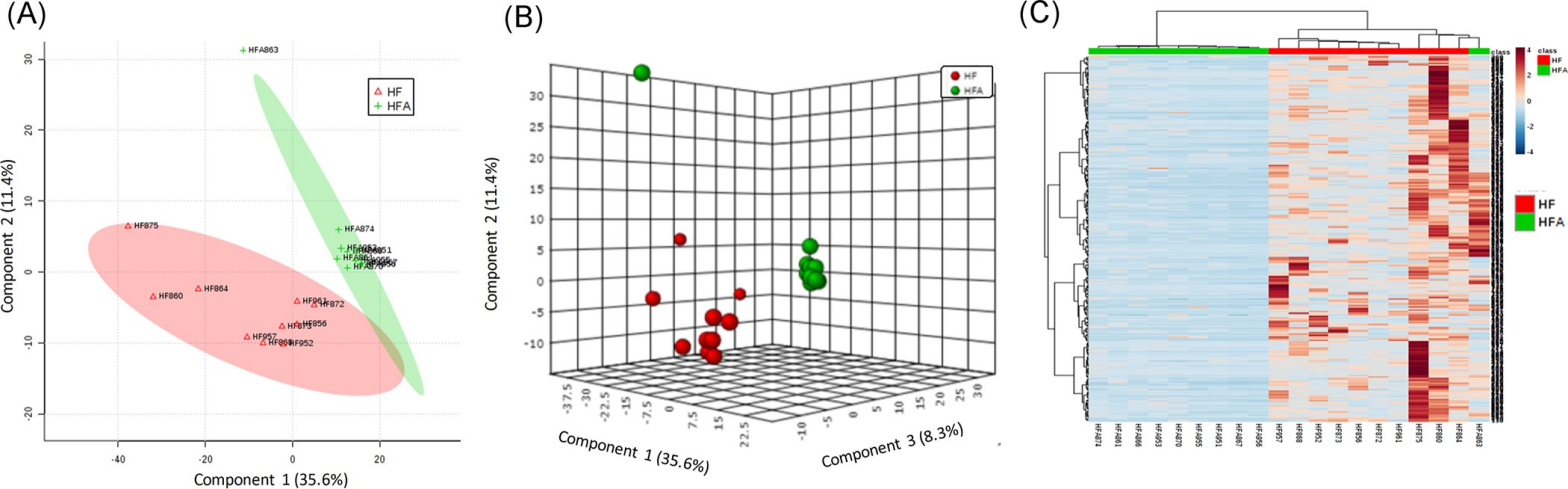
Comparison of colon mucosal proteins in high fat (HF) versus apple-supplemented high fat (HFA) groups. A: Partial least-squares-discriminant analysis (PLS-DA) in two components discriminated HF from HFA groups and explained 47% of the total variance. B: PLS-DA in three components discriminated HF from HFA groups and explained 55% of the total variance. C: Heat map built with the 229 significant proteins (non-parametric Wilcoxon rank-sum test) and FDR cutoff of 0.05. Dark color indicates higher abundance.

Regarding proteomic profile in colonic mucosal cells, the PLS-DA in two components discriminated HF from HFA groups and explained 47% of the total variance (Fig 7A), while PLS-DA in three components discriminated HF from HFA groups and explained 55% of the total variance (Fig 7B). Non-parametric test (Wilcoxon rank-sum test) allowed to identify 229 host proteins differentially expressed in HF and HFA (not shown). These mouse proteins were analyzed using DAVID to generate the functional annotation chart, which identified the biological processes in which such proteins are involved (S1 Supporting Information). Likewise, the hierarchical cluster analysis (Fig 7C), illustrates the relatively higher expression of these proteins in HF group. Interestingly, several of the upregulated proteins in HF group have a biological function in cell adherence and cell-cell junction processes; e.g. epithelial cell adhesion molecule (Epcam) (UniProt accession # Q99JW5), frequently overexpressed in colon cancer [41], and contribute to the formation of intestinal barrier [42]. These results are intriguing considering that HF group had increased translocation of the gut microbiota-derived LPS. In general, further studies are needed to investigate the role of apple supplementation on the modulation of cell junction proteins in colon.

**Fig 8:**
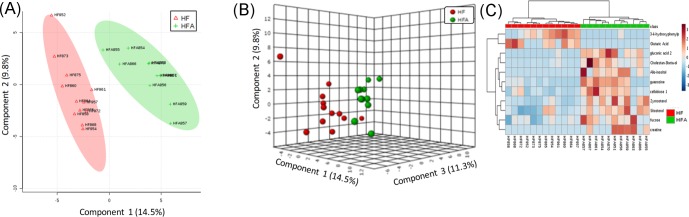
Comparison of fecal metabolites in high fat (HF) versus apple-supplemented high fat (HFA) groups. (A) Partial least-squares-discriminant analysis (PLS-DA) in two components discriminated HF from HFA groups and explained 24.3% of the total variance. (B) PLS-DA in three components discriminated HF from HFA groups and explained 35.6% of the total variance. (C) Heat map built with the 11 significant metabolites (non-parametric Wilcoxon rank-sum test) and FDR cutoff of 0.05. Dark color indicates higher abundance.

Metabolomics analysis resulted in 123 tentatively identified fecal metabolites with 0.05 as adjusted p-value (FDR) cutoff. The non-parametric Wilcoxon rank-sum test resulted in 11 metabolites whose relative abundances were significant (*P* < 0.05) (S1 Supporting Information). The PLS-DA discriminated HF from HFA groups in 2 and 3 components and explained 24.3% and 35.6% of the total variance, respectively (Fig 8A and 8B). Furthermore, a heat map illustrates the relative abundances of the 11 significant metabolites (Fig 8C) shows that eight out of the eleven metabolites were in relative higher abundance in HFA (gluconic acid 2, cholestan-3beta-ol, allo-inositol, guanosine, cellobiose 1, zymosterol, sitosterol, fucose, creatine), while only 3-4-hydroxyphenylpropionic acid and glutaric acid were more abundant in HF group. Among these metabolites, zymosterol and sitosterol are phytosterols derived apples, known to exert LDL-cholesterol lowering properties and other biological effects including anticancer [43,44].

## Conclusions

This work adds valuable information to the literature but there are a number of pitfalls that are important to discuss in order to guide future investigations on the effect of apple supplementation on the gut microbiota and overall health. First, there is great variation in nutritional content (e.g. fiber and pectin concentrations) among different apple cultivars and also between ripeness stages and here we only studied the effect of granny apples at one fixed ripeness stage. Second, fibers and other dietary components affect the gut microbiota at the species level [45] but the 16S rDNA is not discriminative enough to reach the species level. This is important because the variation between subjects involves differences at the species or even at the strain level [46]. Third, the administration of specific bioactive compounds (as opposed to the whole fruit) may have a completely different effect *in vitro* and *in vivo*. For example, Masumoto et al. [4] showed that supplementation of non-absorbable procyanidins led to a decrease in the Firmicutes:Bacteroidetes ratio. Also, the intake of whole apples or clear apple juice has been shown to produce highly contrasting effects on plasma lipids in healthy volunteers [47]. Fourth, the use of sucrose to maintain isocaloric contents in the diets may by itself be responsible for alterations in the intestinal microbial ecosystem [48]. Finally, in this study we decided to include more animals in the HF group based on what is currently known in the literature, which could have been associated with the presence or lack thereof of treatment effect. However, we performed additional analyses using a subset of samples from the HF group and demonstrated that the observed effect of apple supplementation on the gut microbiota was still noticeable even when using as little as five randomly selected samples from each treatment group (see “Beta diversity analyses” above).

In summary, this paper shows that apple consumption has the potential of helping patients with body weight disorders. This study shows that apple supplementation for six weeks has a measurable impact on the abundance of specific fecal bacterial groups *in vivo*, particularly in the presence of a high-fat diet. This change in composition was accompanied by a change in whole communities, biochemical and inflammatory biomarkers in plasma, fecal and host proteins in colon mucosa, as well as fecal metabolites other than SCFAs, which remained similar. These results suggest unique host response signatures driven in part by the differences in the microbiota colonization and contribute to the understanding of microbiome-host interactions with implications in health and diseases, but more studies are needed to explore different apple varieties at different ripeness stages since apple composition (metabolites, fiber, pectins, etc) will differ among cultivars and might influence differently human and animal health.

## Supporting information

S1 Supporting Information (DOCX)
