# Supplementary material for: Apple consumption is associated with a distinctive microbiota, proteomics and metabolomics profile in the gut of Dawley Sprague rats fed a high-fat diet
Source: PLoS One. 2019 Mar 14;14(3):e0212586. doi: 10.1371/journal.pone.0212586 (PMC6417679; doi:10.1371/journal.pone.0212586)
Supplement: S1 Supporting Information — (DOCX) [file pone.0212586.s001.docx]

**Apple consumption is associated with a distinctive microbiota, proteomics and metabolomics profile in the gut of Dawley Sprague rats fed a high-fat diet**

**(SUPPLEMENTARY INFORMATION)**

Jose F. Garcia-Mazcorro^1,2^, Romina Pedreschi^3^, Jialing Yuan^4^, Jorge R. Kawas^5^, Boon Chew^6^, Scot E. Dowd^7^ and Giuliana Noratto^4,6*^

^1^ Research and Development, MNA de México, San Nicolás de los Garza, Nuevo León, México

^2^ Faculty of Veterinary Medicine, Universidad Autónoma de Nuevo León (UANL), General Escobedo, Nuevo León, México

^3^ Escuela de Agronomía, Pontificia Universidad Catolica de Valparaiso, La Palma, Chile

^4^ School of Food Science, Washington State University, Pullman, Washington, USA

^5^ Faculty of Agronomy, UANL, General Escobedo, Nuevo León, México

^6^ Department of Nutrition and Food Science, Texas A&M University, College Station, Texas, USA

^7^ Molecular Research LP, Shallowater, Texas, USA

*Corresponding author.

Email address: gnoratto@tamu.edu (Giuliana Noratto).

**Introduction**

As mentioned in the main text, the aim of this study was to investigate the effect of apple supplementation on the fecal microbiota of rats exposed to a high-fat and a low-fat diet, and to determine how microbiota changes can be related to changes in relevant gut biomarkers and metabolites. Here we include Supplementary Information that may be of value for those readers interested in the topic. This file contains: Source of apples and diet preparation; Proteomic analysis of feces and colonic mucosal cells; Metabolomics analysis in feces; Multivariate analysis of proteomics and metabolomics data; Food intake over time; Analysis at the OTU level; Fig S1. Food intake (in grams, y axis) over time for all treatment groups; Fig. S2 Comparison of fecal proteins in high fat (HF) versus apple-supplemented high fat (HFA) groups; Fig. S3. Colonic mucosal proteins differentially expressed in HF and HFA groups; Table S1. List of primers used for quantitative real-time PCR analyses; Table S2. List of primers used to measure mRNA levels of relevant biomarkers; Table S3. List of the ten more significant OTUs that were different in at least one treatment group; Table S4. Results (median, minimum-maximum) of short-chain fatty acids (SCFAs) in feces (mmol/mg); Table S5. mRNA levels of biomarkers involved in inflammation, cellular stress, tight junction and barrier function in colonic mucosal cells; Table S6. Fecal proteins differentially expressed HF and HFA groups; Table S7. Colon mucosal proteins differentially expressed HF and HFA groups. Table S8. Metabolites in feces that were significantly different between control and supplemented rats within the HF group.

**Materials and methods**

**Source of apples and diet preparation**

Granny Smith apples grown in Washington State were purchased ripened from a local grocery market (Walmart) in Pullman, WA, USA. Apple cores were removed, and apple slices containing peels and flesh were blanched at 90 ºC for 5 min with 0.5 % w/w sodium phytate, homogenized in a blender and freeze dried. The freeze-dried apple powder was kept at -20 ºC for preparation of diet every other day and animal feeding every other day. Freeze dried Granny Smith apples (27.57 ± 0.16 % moisture) contained 0.98 ± 0.12 mg gallic acid equivalents (GAE)/g dry weight (DW) as total extractable phenolics, 4.10 ± 0.46 mg proanthocyanidins/g DW as non-extractable phenolics (analyzed as reported by Condezo-Hoyos et al. [1]), 25.9 ± 1.14 % DW of dietary fiber (16.0 ± 1.14 % insoluble fiber and 9.9 ± 0.14 % soluble fiber) (analyzed using Megazyme kit, Wicklow, Ireland).

Diet was prepared in house by dissolving the agar in distilled water and mixing with powdered ingredients. Diet mixture was solidified at 4º C for 2 h, cut into blocks of approximately 2×2 cm and stored at 4º C until use. Sucrose was used in HFD and LFD in replacement of apple to maintain isocaloric contents between HFD and HFA and between LF and LFA.

**Proteomic analysis of feces and colonic mucosal cells**

Proteins extracted with RIPA buffer (500 ng) from fecal and colonic mucosal cells samples were quantified with a Quant-iT Protein Assay Kit (ThermoFisher Scientific, USA) in compliance with the manufacturer’s protocol and subjected to trypsinization before injection. Disulfide bonds in protein extracts were reduced using 100 mM dithiothreitol (DTT) at a ratio of 1:10 DTT/sample volume and incubated at 50 °C for 45 min. Cysteine bonds were then alkylated with 200 mM iodoacetamide at the same volume ratio for 20 min at room temperature. Protein was digested with trypsin (G-Biosciences, St. Louis, MO, USA) at a 1:50 ratio of trypsin/protein, and incubated at 37 °C for 12 h for high resolution nano-HPLC tandem mass spectrometry analysis. Peptide samples were subjected to Thermo ScientificTM Orbitrap FusionTM TribridTM with an Easy-nLCTM 1 000 ultra-high pressure LC on a Thermo ScientificTM PepMap 100 C18 column (2 μm, 50 μm x 15 cm). The peptides were separated over 115 min gradient eluted at 400 ƞL/min with 0.1% FA in water (solvent A) and 0.1% FA in acetonitrile (solvent B) (5-30% B in 85 min, followed by 30-50% B over 10 min and 50-97% B over 10 min). The run was completed by holding a 97% B for 10 min. MS1 data was acquired on an Orbitrap Fusion mass spectrometry using a full scan method according to the following parameters: scan range 400-1 500 m/z, Orbitrap resolution 120 000; AGC target 400 000; and maximum injection time of 50 ms. MS2 data were collected using the following parameters: rapid scan rate, HCD collision energy 35%, 1.6 m/z isolation window, AGC 2 000 and maximum injection time of 50 ms. MS2 precursors were selected for a 3 s cycle. The precursors with an assigned monoisotopic m/z and a charge state of 2-7 were interrogated. The precursors were filtered using a 60 s dynamic exclusion window. MS/MS spectra were searched using Thermo ScientificTM Proteome DiscovererTM software version 2.0 with SEQUEST® against Uniprot database. Fasta files were prepared based on main bacteria identified on 16S rRNA sequencing for fecal microbiota data, or based on Mus musculus (host) for colon mucosal cells data. Precursor and fragment mass tolerances were set to 10 ppm and 0.8 Da respectively and allowing up to two missed cleavages. Static modification used was carbamidomethylation (C). Protein relative quantitation was achieved by extracting peptide areas with the Proteome Discoverer (PD) 2.0 (Thermo Scientific, San Jose, CA, USA). Three unique peptides per protein were used for the protein quantitation analysis. Data showing FidoCT group confidence as high (q-value < 0.01) indicating that there is less than 1% chance that protein was falsely identified, and false discovery rate (FDR) ≤ 0.05 was selected for statistical analysis. Fecal and colon mucosal cells proteomic data showing significance (*P* < 0.05, Wilcoxon rank-sum test) was subjected to partial least-squares discriminant analysis (PLS-DA), a supervised technique to sharpen the discrimination among the treatments according to similar protein expression profiles. Database for Annotation, Visualization and Integrated Discovery (DAVID) v6.8 was used for bioinformatics and pathway analysis [2,3] on significant colonic mucosal proteomic data (*P* < 0.05) to link protein with biological process and/or disease.

**Metabolomics analysis in feces**

Defined amounts of fecal matter was suspended in 300 μL methanol. After adding 1.5 μg of the surrogate standard ribitol, the material was continued extracted by shaking at room temperature for 15 min (Vortex), sonication for 20 min (Branson 450 sonication bath), and shaking for 15 min at 35 °C and 1200 rpm (Eppendorf Thermomixer). The extracts were then centrifuged for 10 min at 21,000 g, and the supernatants transferred into new vial. The residue was extracted a second time in the same manner but with 500 μL of a solvent mixture containing methanol, 2-propanol, and water at a ratio of 5:2:2. The debris was again removed by centrifugation for 10 min at 21,000 g, and the supernatants combined with the products of first extraction. The combined extracts were dried in vacuum. Dry residues were suspended in 5 μL O-methoxylamine hydrochloride (40 mg mL-1 in pyridine, both from Sigma) and incubated for 90 min at 30°C and 1000 rpm (Eppendorf Thermomixer). Subsequently, samples were derivatized with 45 μL of MSTFA with 1% TMCS (Thermo-Pierce cat.-no.TS-48915) for 30 min at 37°C and 1000 rpm (Eppendorf Thermomixer). A mixture of fatty acid methyl esters was added to each sample prior to injection. Gas chromatography-mass spectroscopy analysis was performed using a Pegasus 4D time-of-flight mass spectrometer (LECO) equipped with a Gerstel MPS2 autosampler and an Agilent 7890A oven. The derivatization products were separated on a 30 m, 0.25 mm i.d., 0.25 μm df Rxi-5Sil® column (Restek) with an IntegraGuard® pre-column using ultrapure He at a constant flow of 1 mL min-1 as carrier gas. The linear thermal gradient started with a one-minute hold at 50 °C, followed by a ramp to 330 °C at 20 °C min-1. The final temperature was held for 5 min prior to returning to initial conditions. Mass spectra were collected at 17 spectra s-1. The injection port was held at 250 °C, and 2 μL of the sample were injected at an appropriate split ratio. Peak identification was conducted using Fiehn primary metabolite library [4]. Peak alignment and spectrum comparisons were carried out using the Statistical Compare feature of the ChromaTOF® software (LECO). The surrogate standard ribitol and the initial matter weight were used for normalization. Primary metabolite derivatization and gas chromatography time-of-flight mass spectrometry analysis was performed by the Laboratory for Cellular Metabolism and Engineering (LCME) at WSU.

**Multivariate analysis of proteomics and metabolomics data**

Statistical analyses were performed on normalized data using MetaboAnalyst 3.0 [5]. Principal component analysis (PCA), an unsupervised technique, was carried out as a first exploration of the data. Partial least-squares discriminant analysis (PLS-DA) analysis, a supervised technique, was carried out to sharpen the discrimination among the treatments according to similar protein/metabolite expression profiles. Only proteins/metabolites that were significant (*P* < 0.05) between experimental groups using the non-parametric Wilcoxon ram sum test and false discovery rate (FDR) cutoff of 0.05 were used for hierarchical clustering analysis using the Euclidean distance. Spearman’s correlations matrices featuring variables identified as significant (*P* < 0.05), were performed using R studio 3.4.0.

**Food intake over time**

Food intake over time was similar among all treatment groups with the exception of the LF group that showed a wider variation in food intake over time (Figure S1).

**Analysis at the OTU level**

As mentioned in the main text, the analysis of relative proportions of 16S sequences assigned as whole groups or taxa often misses potential differences at the OTU level, a topic of great relevance in microbial ecology [6]. The statistical analysis of OTU abundances yielded a total of 44 OTUs that were significantly different in at least one of the four treatment groups (Table S3). From these, only 7 OTUs were “original” OTUs; the rest (37 OTUs) were new OTUs that were created using sequences that did not match the reference sequence file, as described by the pipeline published by Rideout et al. [7]. Despite the controversy about OTU picking approaches [8], this result is very important because these differences would not have been noticed if one would only apply a closed reference approach. Interestingly, several OTUs were higher or lower in abundance in at least one of the LF groups (Table S3), despite the lack of significance for all the taxonomic groups with the exception of *Blautia*.

**Supplementary Figures**


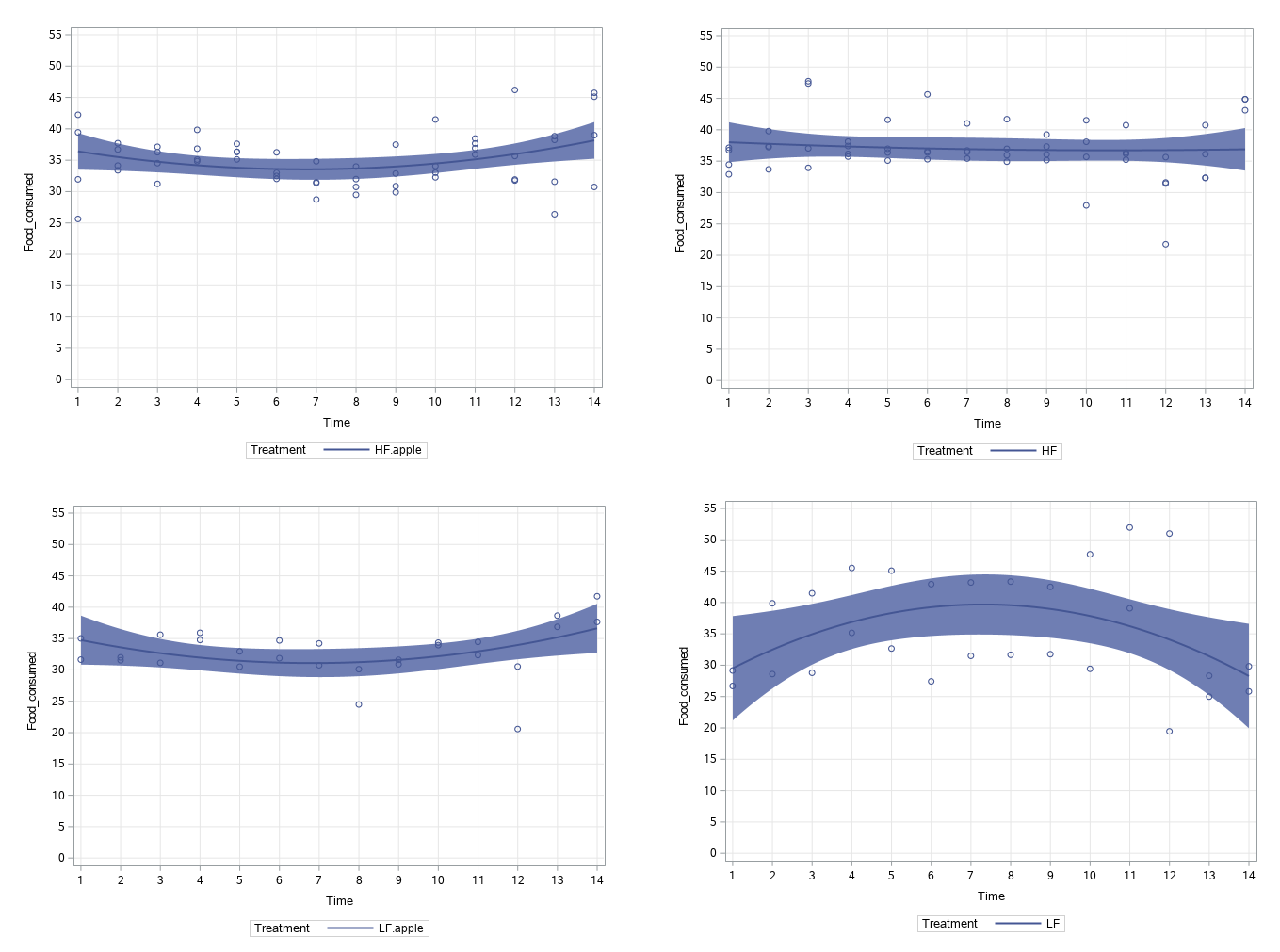


**Supplementary Figure S1**. **Food intake (in grams, y axis) over time for all treatment groups.** This figure clearly shows that the pattern and variability of food intake in the LF group was different from the rest.


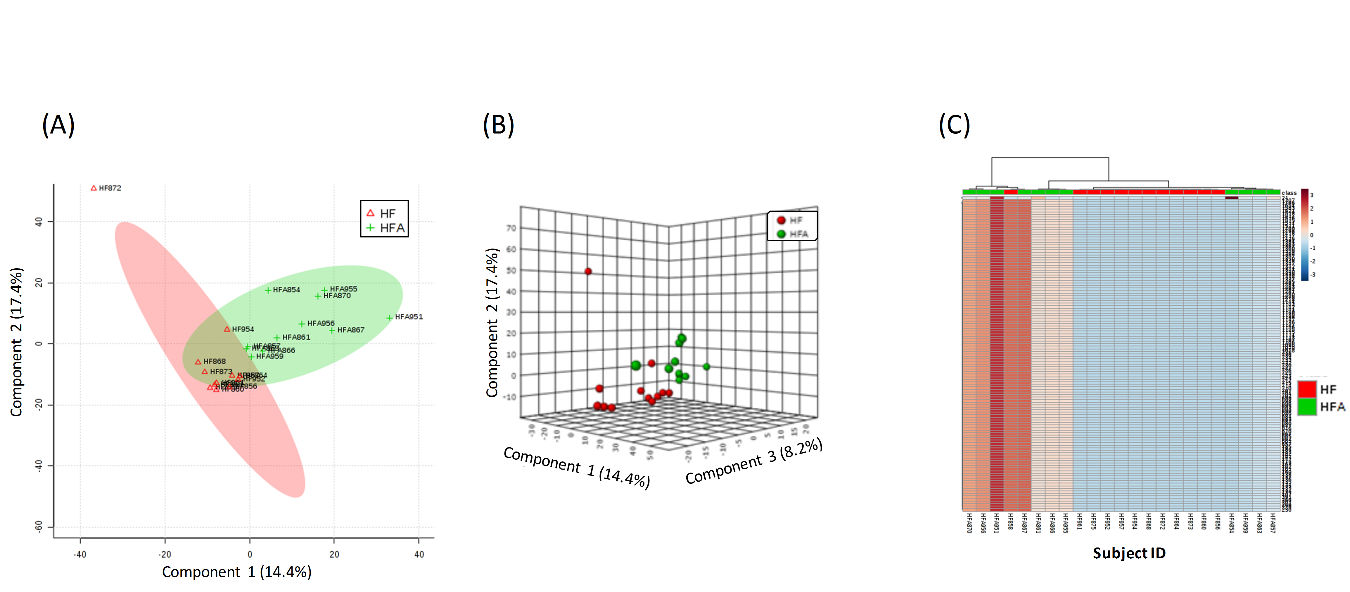


**Supplementary Figure S2**. **Comparison of fecal proteins in high fat (HF) versus apple-supplemented high fat (HFA) groups.** (A) Partial least-squares-discriminant analysis (PLS-DA) in two components discriminated HF from HFA groups and explained 31.8% of the total variance. (B) PLS-DA in three components discriminated HF from HFA groups and explained 40% of the total variance. (C) Heat map built with the 124 proteins significant with the non-parametric t-test (Wilcoxon rank-sum test) and FDR cutoff of 0.05. Dark color indicates higher abundance.

**Supplementary Figure S3. Colonic mucosal proteins differentially expressed in HF and HFA groups.** Bars represent the protein count involved in biological processes (Y axis).

**Supplementary Tables**

**Table S1.** **List of primers used for quantitative real-time PCR analyses.**

| Target | Primers (5’-3’) | Reference |
| --- | --- | --- |
| Firmicutes | TGAAACTYAAAGGAATTGACG  ACCATGCACCACCTGTC | Bacchetti De Gregoris et al. [9] |
| Ruminococcaceae | ACTGAGAGGTTGAACGGCCA  CCTTTACACCCAGTAAWTCCGGA | Garcia-Mazcorro et al. [10] |
| *Faecalibacterium* spp. | GAAGGCGGCCTACTGGGCAC  GTGCAGGCGAGTTGCAGCCT | Garcia-Mazcorro et al. [10] |
| *L. acidophilus* | GCAGATCGCATGATCAGCTTATA  TCAGTCTCTCAACTCGGCTATG | Firmesse et al. [11] |
| *Turicibacter* | CAGACGGGGACAACGATTGGA  TACGCATCGTCGCCTTGGTA | Suchodolski et al. [12] |
| *Akkermansia* | CAGCACGTGAAGGTGGGGAC  CCTTGCGGTTGGCTTCAGAT | Collado et al. [13] |
| Enterobacteriaceae | CATTGACGTTACCCGCAGAAGAAGC  CTCTACGAGACTCAAGCTTGC | Bartosch et al. [14] |
| *E. coli* | CATGCCGCGTGTATGAAGAA  CGGGTAACGTCAATGAGCAAA | Huijsdens et al. [15] |
| *B. bifidum* | CCACATGATCGCATGTGATTG  CCGAAGGCTTGCTCCCAAA | Matsuki et al. [16] |
| Bacteroidetes | GGARCATGTGGTTTAATTCGATGAT  AGCTGACGACAACCATGCAG | Guo et al. [17] |
| *B. fragilis* | CTGAACCAGCCAAGTAGCG  CCGCAAACTTTCACAACTGACTTA | Liu et al. [18] |
| *Bacteroides/Prevotella* | GAGAGGAAGGTCCCCCAC  CGCTACTTGGCTGGTTCAG | Layton et al. [19] |

**Table S2.** **List of primers used to measure mRNA levels of relevant biomarkers.**

| Target | Forward primer (5’ to 3’) | Reverse primer (5’ to 3’) |
| --- | --- | --- |
| IL-1β* | TCGCTCAGGGTCACAAGAAA | CATCAGAGGCAAGGAGGAAAAC |
| TNF-α* | AAATGGGCTCCCTCTCATCAGTTC | TCTGCTTGGTGGTTTGCTACGAC |
| NF-kB | GGA TGG TGA GGT CAC TCT | TCCTGAACTCCAGCACTCTCTTC |
| ATF4* | GAGCTTCCTGAACAGCGAAGTG | TGGCCACCTCCAGATAGTCATC |
| CHOP* | CCTAGCTTGGCTGACAGAGG | CTGCTCCTTCTCCTTCATGC |
| PG* | ATGAAGACCATTTACTTTG | CGGTTCCTCTTGGTGTTCATCAAC |
| ZO-1* | ACCCGAAACTGATGCTGTGGATAG | AAATGGCCGGGCAGAACTTGTGTA |
| Occ* | ATGTCCGGCCGATGCTCTC | TTTGGCTGCTCTTGGGTCTGTAT |
| F4/80* | TGACAACCAGACGGCTTGTG | CAGGCGAGGAAAAGATAGTGT |
| MCP-1 | CAAGCAGAAGTGGGTTCAGGAT | TCTTCGGAGTTTGGGTTTGC |
| VCAM-1 | GTCACGGTCAAGTGTTTGGC | AGATCCGGGGGAGATGTCAA |
| RPL19* | GAAGGTCAAAGGGAATGTGTTCA | CCTTGTCTGCCTTCAGCTTGT |

IL-1 β: interleukin-1β; TNF-α: tumor necrosis factor alpha; NF-*k*B: nuclear factor kappa B; ATF4: activating transcription factor 4; CHOP: CCAAT/enhancer binding protein homologous protein; PG: proglucagon; ZO-1: zonula occludens-1; Occ: occluding; F4/80: macrophage F4/80 receptor; MCP-1: monocyte chemoattractant protein-1; VCAM-1: vascular cell adhesion molecule 1; RPL19: ribosomal protein L19. *Primer sequences were obtained from Pachikian et al. [20]. Primers for NF-kB, MCP-1 and VCAM-1 were designed using the Primer Express software (Applied Biosystems).

**Table S3.** **List of the ten more significant OTUs that were different in at least one treatment group.**

| OTU with Taxonomy | Corrected *P* value | Highlights |
| --- | --- | --- |
| NR775/Desulfovibrionaceae | 0.0001 | Higher in LF diet, particularly without apple supplementation |
| NCU100792/Unassigned Bacteroidetes* | 0.0001 |  |
| NR86/*Bacteroides* | 0.0003 |  |
| NR838/Clostridiales | 0.001 |  |
| NCU50910/Clostridiales | 0.002 |  |
| NR602/*Butyricimonas* | 0.003 |  |
| 441468/Clostridiales | 0.002 | Higher in LF diet, particularly with apple supplementation |
| NCU13229/Ruminococcaceae | 0.003 | Higher in LF diet with apple supplementation |
| NR563/RF32 (order within the Alphaproteobacteria) | 0.002 | Higher in HF diet, particularly without apple supplementation |
| NR212/Ruminococcus | 0.004 | Higher in HF diet without apple supplementation |

NR=New reference OTU, NCU=New Cleaned-Up OTU. *This OTU was catalogued as Unassigned by QIIME. The proposed taxonomy is based on a search using the classifier option of the Ribosomal Database Project.

**Table S4. Results (median, minimum-maximum) of short-chain fatty acids (SCFAs) in feces (mmol/mg).**

|  | HF (n=12) | HF apple (n=13) | LF (n=5) | LF apple (n=6) |
| --- | --- | --- | --- | --- |
| Sodium butyrate | 0.15 (0.06-0.63) | 0.26 (0.12-0.41) | 0.12 (0.11-0.28) | 0.13 (0.08-0.21) |
| Sodium propionate | 0.09 (0.07-0.62) | 0.10 (0.08-0.61) | 0.08 (0.05-0.17) | 0.09 (0.09-0.10) |
| Acetic acid | 15.6 (10.9-20.9) | 17.9 (13.5-27.3) | 17.6 (9.1-22.3) | 17.4 (15.5-20.7) |

**Table S5. mRNA levels of biomarkers involved in inflammation, cellular stress, tight junction and barrier function in colonic mucosal cells.**

| **Genes** | **mRNA levels/β-actin mRNA** | **HF** | **HFA** | **Fold change HFA/HF** | ***P* value** |
| --- | --- | --- | --- | --- | --- |
| Inflammation/ Cellular stress | TGF-β1 | 0.9 (0.09-2) | 3(0.3-8) | 3.0 | 0.0157 |
|  | TNF-α | 1.2 (0.15-4.6) | 2.5 (0.6-7) | 1.9 | 0.1290 |
|  | PPARγ | 1.6 (0.09-9.4) | 3.5 (0.7-15) | 1.5 | 0.2727 |
|  | ATF4 (mean, SEM) | 2.8 (0.1-9.1) | 3.6 (1.1-9.9) | 1.3 | 0.2959 |
|  | VCAM-1 | 1.9 (0.1-7.1) | 2.4 (0.1-14) | 1.9 | 0.1499 |
| Tight junction and barrier function | OCC (mean, SEM) | 0.3 (0.06-1.3) | 1.1 (0.05-2.3) | 2.4 | 0.1135 |
|  | ZO-1 | 2.1 (1.3-3.2) | 1.8 (1.5-3.1) | 0.9 | 0.2959 |
|  | CHOP (mean, SEM) | 2.6 (0.08-9) | 2.5 (0.5-11) | 1.3 | 0.4701 |

Data are median (min-max). Data analyzed with Mann-Whitney test, and outlier detection were performed with GraphPad Prism 6.0.

**Table S6. Fecal proteins differentially expressed HF and HFA groups.**

| **Protein names** | **Count*** | **Organism** |
| --- | --- | --- |
| Transcription elongation factor GreA | 27 | Order Clostridiales |
| Transcription elongation factor GreA | 18 | Lachnospiraceae bacterium |
| Transcription elongation factor GreA | 15 | *Eubacterium* |
| Transcription elongation factor GreA | 10 | *Roseburia* |
| Transcription elongation factor GreA | 9 | *Blautia* |
| Transcription elongation factor GreA | 6 | *Clostridium* |
| Transcription elongation factor GreA | 6 | *Coprococcus* |
| Transcription elongation factor GreA | 6 | *Oribacterium* |
| Transcription elongation factor GreA | 5 | *Butyrivibrio* |
| Transcription elongation factor GreA | 5 | *Dorea* |
| Transcription elongation factor GreA | 9 | *Ruminococcus* |
| Transcription elongation factor GreA | 2 | *Hungatella* |
| Flagellin | 1 | *Eubacterium plexicaudatum* ASF492 |
| Transcription elongation factor GreA | 1 | Tyzzerella nexilis DSM 1787 |
| Transcription elongation factor GreA | 1 | *Eubacterium rectale* DSM 17629 |
| Transcription elongation factor GreA | 1 | *Agathobacter rectalis* (*Eubacterium rectale*) |
| Transcription elongation factor GreA | 1 | *Anaerostipes* sp. CAG:276 |
| Transcription elongation factor GreA | 1 | *Catonella morbi* ATCC 51271 |
| Transcription elongation factor GreA | 1 | *Johnsonella ignava* ATCC 51276 |
| Transcription elongation factor GreA | 1 | *Marvinbryantia formatexigens* DSM 14469 |
| Transcription elongation factor GreA | 1 | *Stomatobaculum longum* |

* Count represents the number of organisms belonging to the same order/class/family identified as the source of specific protein.

**Table S7. Colon mucosal proteins differentially expressed HF and HFA groups.**

| Biological Process | Count* | FDR |
| --- | --- | --- |
| Cell-cell junction | 12 | 0.018733 |
| Myelin sheath | 12 | 0.012167 |
| Spliceosomal complex | 12 | 4.13E-04 |
| Chaperone | 13 | 6.47E-04 |
| Spliceosome | 13 | 6.09E-05 |
| Actin-binding | 15 | 3.26E-04 |
| mRNA splicing | 15 | 1.80E-04 |
| Focal adhesion | 16 | 0.037976 |
| Intracellular ribonucleoprotein complex | 16 | 0.003499 |
| Protein complex binding | 16 | 0.036776 |
| RNA recognition motif domain | 16 | 6.18E-05 |
| RNA splicing | 16 | 1.97E-04 |
| Nucleotide-binding, alpha-beta plait | 17 | 7.50E-05 |
| Actin binding | 19 | 1.78E-04 |
| Cell-cell adhesion | 19 | 9.88E-09 |
| mRNA processing | 20 | 9.24E-06 |
| Nucleolus | 27 | 0.002519 |
| RNA-binding | 28 | 9.04E-08 |
| RNA binding | 29 | 2.58E-04 |
| Cell-cell adherens junction | 32 | 1.25E-17 |
| Cadherin binding involved in cell-cell adhesion | 33 | 2.74E-19 |
| Cytoskeleton | 35 | 2.89E-06 |
| Isopeptide bond | 36 | 3.10E-08 |
| Methylation | 36 | 3.79E-08 |
| Ubiquitin conjugation | 39 | 1.34E-04 |
| Cytosol | 41 | 0.012451 |
| Poly(A) RNA binding | 69 | 1.21E-27 |
| Coiled coil | 73 | 1.23E-09 |
| Protein binding | 79 | 0.004755 |
| Extracellular exosome | 84 | 3.86E-17 |
| Nucleus | 107 | 8.97E-06 |
| Acetylation | 129 | 5.47E-49 |
| Cytoplasm | 136 | 2.01E-15 |
| Phosphoprotein | 178 | 1.59E-40 |

*Indicates the number of identified proteins involved in the biological process. FDR: False Discovery Rate.

**Table S8. Metabolites in feces that were significantly different between control and supplemented rats within the HF group.**

| Metabolite | *P* value |
| --- | --- |
| Gluconic acid 2 | 8.57E-05 |
| Zymosterol | 9.28E-05 |
| 3-4-hydroxyphenylpropionic acid | 0.000226 |
| Guanosine | 0.000503 |
| Sitosterol | 0.000789 |
| Glutaric Acid | 0.000831 |
| Cellobiose 1 | 0.000887 |
| Allo-inositol | 0.000976 |
| Cholestan-3beta-ol | 0.001836 |
| Fucose | 0.002119 |
| Creatine | 0.002892 |

**References**

1. Condezo-Hoyos L, Mohanty IP, Noratto GD. Assessing non-digestible compounds in apple cultivars and their potential as modulators of obese faecal microbiota in vitro. Food Chem. 2014;161:208-215. doi: 10.1016/j.foodchem.2014.03.122.

2. Huang da W, Sherman BT, Lempicki RA. Systematic and integrative analysis of large gene lists using DAVID bioinformatics resources. Nat Protoc. 2009;4(1):44-57.

3. Huang da W, Sherman BT, Lempicki RA. Bioinformatics enrichment tools: paths toward the comprehensive functional analysis of large gene lists. Nucleic Acids Res. 2009;37(1):1-13.

4. Kind T, Wohlgemuth G, Lee DY, Lu Y, Palazoglu M, Shahbaz S, Fiehn O. FiehnLib: mass spectral and retention index libraries for metabolomics based on quadrupole and time-of-flight gas chromatography/mass spectrometry. Anal Chem. 2009;81(24):10038-10048.

5. Xia J, Sinelnikov IV, Han B, Wishart DS. MetaboAnalyst 3.0 -- making metabolomics more meaningful. Nucl Acids Res. 2015;43(W1):W251-257.

6. Koeppel AF, Wu M. Surprisingly extensive mixed phylogenetic and ecological signals among bacterial Operational Taxonomic Units. Nucleic Acids Res. 2013;41(10):5175.5188.

7. Rideout JR, He Y, Navas-Molina JA, Walters WA, Ursell LK, Gibbons SM, et al. Subsampled open reference clustering creates consistent, comprehensive OTU definitions and scales to billions of sequences. PeerJ 2014;2:e545.

8. Westcott SL, Schloss PD. De novo clustering methods outperform reference-based methods for assigning 16S rRNA gene sequences to operational taxonomic units. PeerJ 2015;3:e1487.

9. Bacchetti De Gregoris T, Aldred N, Clare AS, Burgess JG. Improvement of phylum- and class-specific primers for real-time PCR quantification of bacterial taxa. J Microbiol Meth. 2011;86:351-356.

10. Garcia-Mazcorro JF, Suchodolski JS, Jones KR, Clark-Price SC, Dowd SE, Minamoto Y, Markel M, Steiner JM, Dossin O. Effect of the proton pump inhibitor omeprazole on the gastrointestinal microbiota of healthy dogs. FEMS Microbiol Ecol. 2012;80:624-636.

11. Firmesse O, Mogenet A, Bresson JL, Corthier G, Furet JP. Lactobacillus rhamnosus R11 consumed in a food supplement survived human digestive transit without modifying microbiota equilibrium as assessed by real-time polymerase chain reaction. J Mol Microbiol Biotechnol. 2008;14:90-99.

12. Suchodolski JS, Markel ME, Garcia-Mazcorro JF, Unterer S, Heilmann RM, Dowd SE, et al. The fecal microbiome in dogs with acute diarrhea and idiopathic inflammatory bowel disease. PLoS ONE 2012;7(12):e51907.

13. Collado MC, Derrien M, Isolauri E, de Vos WM, Salminen S. Intestinal integrity and *Akkermansia muciniphila*, a mucin-degrading member of the intestinal microbiota present in infants, adults, and the elderly. Appl Environ Microbiol. 2007;73:7767-7770.

14. Bartosch S, Fite A, Macfarlane GT, McMurdo ME. Characterization of bacterial communities in feces from healthy elderly volunteers and hospitalized elderly patients by using real-time PCR and effects of antibiotic treatment on the fecal microbiota. Appl Environ Microbiol. 2004;70:3575-3581.

15. Huijsdens XW, Linskens RK, Mak M, Meuwissen SG, Vandenbroucke-Grauls CM, Savelkoul PH. Quantification of bacteria adherent to gastrointestinal mucosa by real-time PCR. J Clin Microbiol. 2002;40:4423-4427.

16. Matsuki T, Watanabe K, Tanaka R, Oyaizu H. Rapid identification of human intestinal bifidobacteria by 16S rRNA-targeted species- and group-specific primers. FEMS Microbiol Lett. 1998;167:113–121.

17. Guo X, Xia X, Tang R, Zhou J, Zhao H, Wang K. Development of a real-time PCR method for Firmicutes and Bacteroidetes in faeces and its application to quantify intestinal population of obese and lean pigs. Lett Appl Microbiol. 2008;47:367-373.

18. Liu C, Song Y, McTeague M, Vu AW, Wexler H, Finegold SM. Rapid identification of the species of the *Bacteroides fragilis* group by multiplex PCR assays using group- and species-specific primers. FEMS Microbiol Lett. 2003;222:9-16.

19. Layton A, McKay L, Williams D, Garrett V, Gentry R, Sayler G. Development of *Bacteroides* 16S rRNA gene TaqMan-based real-time PCR assays for estimation of total, human, and bovine fecal pollution in water. Appl Environ Microbiol. 2006;72: 4214-4224.

20. Pachikian BD, Neyrinck AM, Deldicque L, De Backer FC; Catry E, Dewulf EM, Sohet FM, Bindels LB, Everard A, Francaux M, Guiot Y, Cani PD, Delzenne NM. Changes in intestinal bifidobacteria levels are associated with the inflammatory response in magnesium-deficient mice. J Nutr. 2010;140(3):509-514.
